# Low Prevalence of Antibodies Against *Toxoplasma gondii* in Chinese Populations

**DOI:** 10.3389/fcimb.2020.00302

**Published:** 2020-06-23

**Authors:** Shilin Xin, Ruijing Su, Nan Jiang, Longxian Zhang, Yurong Yang

**Affiliations:** College of Animal Science and Veterinary Medicine, Henan Agricultural University, Zhengzhou, China

**Keywords:** *Toxoplasma gondii*, seroepidemiology, risk factors, human, modified agglutination test, China

## Abstract

*Toxoplasma gondii* has been found to infect almost all warm-blooded animals, including humans. In this study, a total of 3,275 human serum samples were collected from hospitals in five provinces of China. About 5.13% (168/3,275) (95% CI, 4.42–5.94) of the serum samples tested positive for *T. gondii* IgG antibody by a modified agglutination test (MAT) (cut-off: 1:20). Significant associations were detected between geographic location (OR = 1.763), age (OR = 3.072), infertility in women (OR = 2.4409) and *T. gondii* infection in humans (*p* < 0.05). To minimize infection, citizens need to be informed about the best practices for toxoplasmosis prevention, including eating well-cooked meat, drinking boiled water, washing vegetables and fruits, and being careful during contact with cats.

## Introduction

*Toxoplasma gondii* is a ubiquitous protozoan parasite that is estimated to infect one-third of the human population worldwide. It can infect many species of warm-blooded animals, and is a significant zoonotic and veterinary pathogen. Members of the felid family, which are the definitive hosts of *T. gondii*, shed oocysts in their feces, and other warm-blooded animals may be intermediate hosts (Weiss and Dubey, 2009; Dubey, 2010; Torrey and Yolken, 2013). There are three primary routes from which people become infected with *T. gondii*: (1) by ingesting uncooked meat containing *T. gondii* tissue cysts; (2) by ingesting water, soils, vegetables and fruits contaminated by the feces of infected felids; and (3) transmission from the mother to the fetus via the placenta during pregnancy (Dubey, 1994; Montoya and Liesenfeld, 2004).

*Toxoplasma gondii* infection can become severe in people with AIDS, cancer, and organ transplants, and is usually asymptomatic in people with healthy immune systems (Dubey, 2010). In pregnant women, primary infection during pregnancy can lead to congenital infection of the fetus and newborn, resulting in severe damage, including stillbirth, miscarriage, and ocular toxoplasmosis (Montoya and Liesenfeld, 2004). Therefore, as a foodborne parasite, *T. gondii* infection has become a major potential public health problem worldwide.

*Toxoplasma gondii* is prevalent in most areas of the world, and people seropositive for *T. gondii* have been found in different regions (Tenter et al., 2000; Sukthana, 2006). The first human case of toxoplasmosis in China was reported in 1964 (Xie, 1964). Furthermore, some nationwide epidemiological surveys on *T. gondii* revealed that the in recent years, seropositive rate has been on the rise (1983: 5.20%, 2001–2004: 7.97%, 2000–2017: 8.22%) (Lv, 2002; Xu et al., 2005; Pan et al., 2017; Dong et al., 2018). The purpose of this study was to further estimate the *T. gondii* infection rate in humans, and to study the effects of geographical, age, gender, pregnancy status, healthy conditions, and nursing on the seroprevalence of this disease.

## Materials and Methods

### Participants and Serum Samples

**Figure 1:**
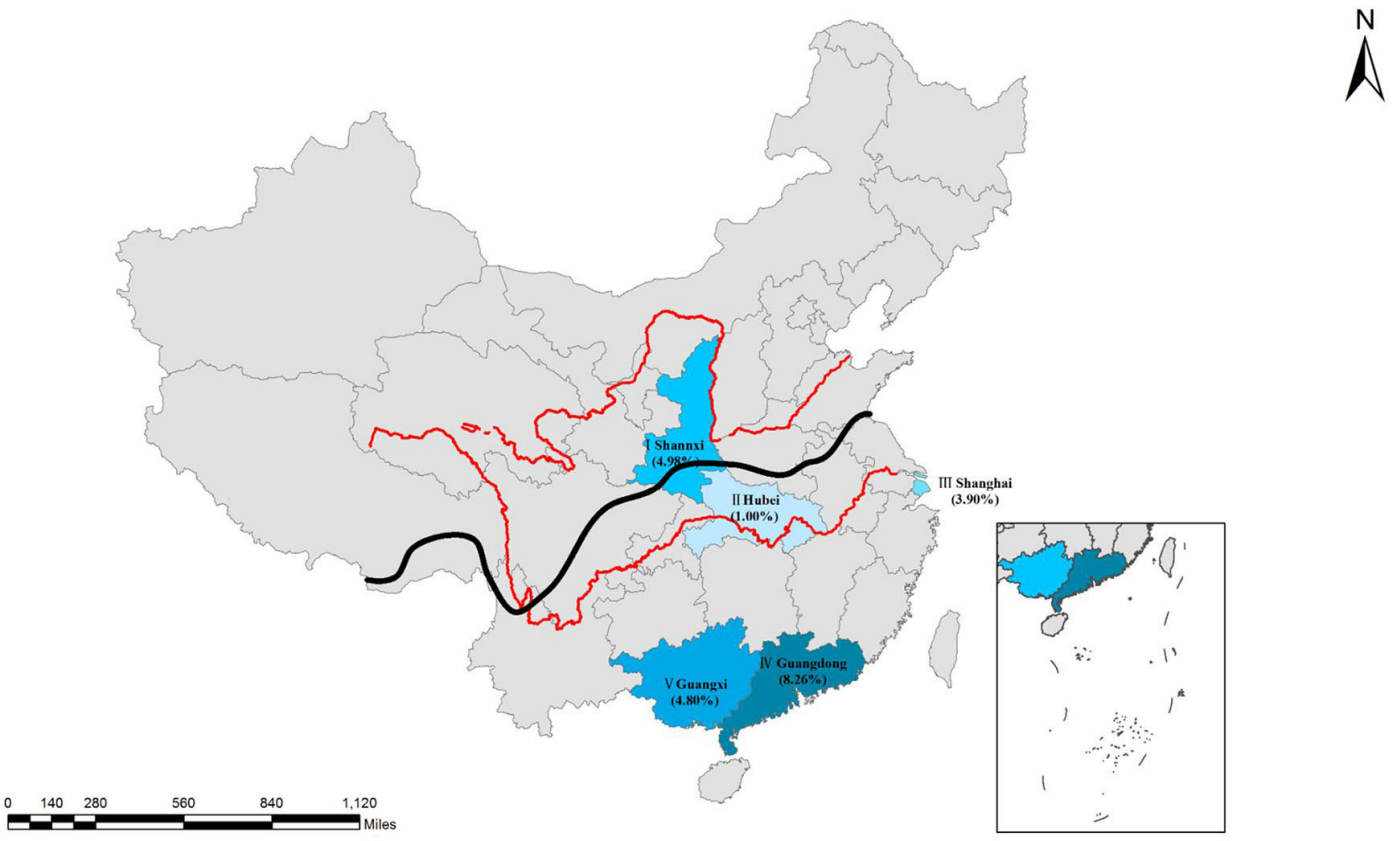
Distribution of seroprevalence of *T. gondii* infection in Chinese populations. I, Shaanxi; II, Hubei; III, Shanghai, IV, Guangdong; V, Guangxi. Qinling Mountains-Huaihe River Line as the south and north of the boundary (The black line is shown in the figure). Red lines are showing the Yellow River and the Changjiang River. Map was adapted from Google earth.

In this study, a total of 3,275 human serum samples were collected from hospitals in the Guangdong, Shanghai, Hubei, Guangxi, and Shaanxi regions of China from May 2018 to August 2019 (Table 1 and Figure 1). These serum samples were transported to the Henan Agricultural University (Zhengzhou, Henan, China) in cooler boxes for a survey of *T. gondii* infection. The age of participants ranged from newborn babies to the elderly, and other basic patient information was also collected. Unfortunately, the gender data information from children (*n* = 1847) were not available.

**Table 1:** Demographic characteristics and seroprevalence of *Toxoplasma gondii* in 3,275 participants.

**Characteristics**		**Samples**	**Positive no. in different titers**										**% (Positive No.)**	**95% Cl**
			**1:20**	**1:40**	**1:80**	**1:160**	**1:320**	**1:640**	**1:1280**	**1:2560**	**1:5120**	**1:10240**		
Location	Guangdong	968	20	12	5	21	3	16	1	–	1	1	8.26(80)	6.68–10.18
	Shanghai	1334	21	3	9	12	3	3	1	–	–	–	3.90(52)	2.98–5.08
	Guangxi	271	9	–	2	1	–	–	1	–	–	–	4.80(13)	2.75–8.11
	Hubei	300	1	–	–	2	–	–	–	–	–	–	1.00(3)	0.20–3.04
	Shaanxi	402	11	1	4	3	–	–	1	–	–	–	4.98(20)	3.20–7.60
Age	0 −14	1847	42	6	10	10	1	3	2	–	1	–	4.06(75)	3.25–5.07
	15 −59	1063	13	4	8	16	5	3	1	–	–	1	4.80(51)	3.66–6.26
	≥60	365	7	6	2	13	–	13	1	–	–	–	11.51(42)	8.60–15.21
	**Total**	3275	62	16	20	39	6	19	4	–	1	1	5.13(168)	4.42–5.94
Gender	Male	224	8	3	–	1	–	1	–	–	1	–	6.25(14)	3.68 −10.30
	Female	988	10	3	2	13	3	4	1	–	–	1	3.74(37)	2.72–5.13
	**Total**	1212	18	6	2	14	3	5	1	–	1	1	4.21(51)	3.21–5.50
Women	Pregnant	751	8	2	2	8	3	2	1	–	–	–	3.46(26)	2.35–5.05
	Infertile	87	2	–	–	3	–	1	–	–	–	1	8.05(7)	3.70–15.94
	**Total**	838	10	2	2	11	3	3	1	–	–	1	3.94(33)	2.80–5.50
Health condition	Health	812	10	2	2	11	3	3	1	–	–	–	3.94(32)	2.79–5.53
	Unhealthy	2129	42	14	16	23	1	15	2	–	1	1	5.40(115)	4.52–6.45
	**Total**	2941	52	16	18	34	4	18	3	–	1	1	5.00(147)	4.27–5.85
Children's diet	Nursing (0-2)	805	17	3	0	5	0	1	1	–	–	–	3.35(27)	2.30–4.86
	Weaning (3-14)	772	16	3	8	4	1	2	–	–	1	–	4.53(35)	3.26–6.26
	**Total**	1577	33	6	8	9	1	3	1	–	1	–	3.93(62)	3.07–5.01

### Ethics Approval and Consent to Participate

This study was carried out in accordance with the recommendations of the guidelines for Using Human and Animals Subjects by the Beijing Association for Science and Technology (SYXK [Beijing] 2007-0023). The sera were collected with the consent of patients or volunteers. Written consent was obtained from parents of all participating juveniles. This study was further approved by the ethics committee of the Henan Agricultural University (China).

### Serological Testing

All of the serum samples were tested for antibodies against *T. gondii* by a modified agglutination test (MAT) (Dubey and Desmonts, 1987). Sera with MAT titers of 1:20 or higher were considered positive for *T. gondii* (Dubey, 2010; Yang et al., 2017). Whole formalin-treated *T. gondii* tachyzoites were obtained from the University of Tennessee Research Foundation (Knoxville, TN, USA; https://utrf.tennessee.edu/). *T. gondii*-positive mouse sera were provided by Dr. J. P. Dubey (Beltsville, ARS, USDA) as reference sera. All of the serum samples were tested at 1:20, after which the dilution was doubled to the maximum titer, and negative and positive controls were included in each plate.

### Statistical Analysis

According the age data, the following categories were made: an older adult group, a young and middle-aged people group, and a children and juveniles group. Data from the five provinces were also sorted geographically into north vs. south, and coastal vs. inland (Figure 1 and Table 2). Furthermore, data were also analyzed and compared in terms of gender, weaning and nursing. The 838 female participants (25–42 years old) were divided into pregnant and infertile categories. In addition, we defined participants who were clinically diagnosed with underlying diseases as unhealthy, and those who were not diagnosed with diseases as healthy (health physical examination). The classification standards of the above groups is summarized in Table 2.

**Table 2:** Seroprevalence and risk factors for *Toxoplasma gondii* in participants tested by modified agglutination test.

**Variable**	**Classification standards**	**Samples**	**Seropositivity (%)**	**Odds ratio (95% Confidence internal)**	***P*–value**
**AGE**					
Older adults	≥ 60 years	365	11.51	3.072 (2.068–4.565)	0.0001^*^
Young and middle-aged people	15–59 years	1063	4.80	1.191 (0.8273–1.714)	0.3975
Children and juveniles	0–14 years	1847	4.06	1	
**CHILDREN'S DIET**					
Weaning	3–14 years	772	4.53	1.368 (0.8199–2.284)	0.2282
Nursing	0–2 years	805	3.35	1	
**GENDER**					
Male	–	224	6.25	1.714 (0.9098–3.227)	0.0980
Female	–	988	3.74	1	
**WOMEN (25–42 YEARS)**					
Infertile	Abortion when embryo is <12 weeks, sterility	87	8.05	2.4409 (1.026–5.801)	0.0374^*^
Pregnant	Embryo is between 12 and 40 weeks	751	3.46	1	
**HEALTH CONDITION**					
Unhealthy	Influenza, respiratory disease, diarrhea, fever, nephrosis, infertile, and nervous system disease	2129	5.40	1.392(0.9325–2.077)	0.1041
Health	Health physical examination personnel and without apparent abnormality	812	3.94	1	
**GEOGRAPHICAL LOCATION**					
South	Shanghai, Guangdong, Guangxi and Hubei	2873	5.15	1.037(0.6424–1.675)	0.8807
North	Shaanxi	402	4.98	1	
Coastal	Shanghai, Guangdong and Guangxi	2573	5.64	1.763(1.126–2.760)	0.0120^*^
Inland	Shaanxi and Hubei	702	3.28	1	

* *Statistically significant*.

Statistical analysis was performed using the GraphPad Prism 6.0 software (GraphPad Software Inc., San Diego, CA, USA). The results were analyzed by the Chi-square or Fisher's exact test and the Monte Carlo test of simulated data to assess the risk factors associated with *T. gondii* infection. A *p-*value of <0.05 was considered to be statistically significant.

## Results

### Seroprevalence of *T. gondii* in Humans and Risk Factor Analysis

In this study, blood from 3,275 participants was evaluated for *T. gondii* infection. The results indicated that 5.13% (168/3,275) (95% CI, 4.42–5.94) of the participants were seropositive for *T. gondii* by MAT, with titers of 1:20 in 62, 1:40 in 16, 1:80 in 20, 1:160 in 39, 1:320 in six, 1:640 in 19, 1:1,280 in four, 1:5,120 in one and 1:10,240 in one across the participants (Table 1).

All of the participants were divided into three age groups. The seroprevalence of *T. gondii* was 4.06% (75/1,847, 95% CI, 3.25–5.07) for 0–14 years old, 4.80% (51/1,063, 95% CI, 3.66–6.26) for 15–59 years old, and 11.51% (42/365, 95% CI, 8.60–15.21) for ≥ 60 years old. Compared with the other two age groups, the prevalence of *T. gondii* infection was higher in the age group ≥ 60 years, and the difference is very significant (*p* < 0.01), with an odds ratio of 3.072 (95% CI, 2.068–4.565) (Tables 1, 2). Meanwhile, within the 0–14 age group, the prevalence of *T. gondii* in the weaning group (3–14 years) (4.53%, 35/772) was higher than that of the nursing group (0–2 years) (3.35%, 27/805), although the difference was not statistically significant (*p* = 0.2282).

In terms of gender, the seroprevalence of *T. gondii* in males (6.25%, 14/224) was higher than that in females (3.74%, 37/988) (*p* = 0.0980). The seroprevalence of *T. gondii* in infertile women (8.05%, 7/87) was higher than in pregnant women (3.46%, 26/751), and the difference was statistically significant (*p* = 0.0374), with an odds ratio of 2.4409 (95% CI, 1.026–5.801). The seroprevalence of *T. gondii* was 5.40% (115/2,129) in the unhealthy group, which was higher than that of the clinically healthy group (3.94%, 32/812) (*p* = 0.1041; Tables 1, 2).

When analyzed by geographic location, the seroprevalence of *T. gondii* varied by region. The seroprevalence of *T. gondii* infection was 5.15% (95% CI, 4.40–6.02) in Southern China, and 4.98% (95% CI, 3.20–7.60) in Northern China (*p* = 0.8807). Additionally, the seroprevalence of *T. gondii* was significantly higher in coastal areas (5.64%, 95% CI, 4.81–6.60) than in inland areas (3.28%, 95% CI, 2.17–4.89) (*p* = 0.0120), with an odds ratio of 1.763 (95% CI, 1.126–2.760) (Tables 1, 2).

## Discussion

The epidemiology of toxoplasmosis has been investigated in many countries, including China. The main detection methods for *T. gondii* include serological tests, PCR, isolation methods, and histopathology. Among them, serological tests are the most sensitive, rapid, and economical (Greiner and Gardner, 2000a,b; Hill et al., 2006; Dubey, 2008; Dard et al., 2016). However, serological tests vary in sensitivity, specificity, and predictive values (Cubas-Atienzar et al., 2019; Khan and Noordin, 2020). The Sabin-Feldman dye test (DT) is the most specific test for *T. gondii*, and it is considered the reference test for human diagnosis according to the World Health Organization (Sabin and Feldman, 1948). However, its main disadvantages are that it is labor-intensive and requires live parasites (dangerous). Thus, it was replaced by other tests in most laboratories (Dubey and Desmonts, 1987; Franck et al., 2008; Cubas-Atienzar et al., 2019; Khan and Noordin, 2020). The MAT was established by Dubey and was considered to be reliable after comparing several serological test methods with animal tissue biopsy results (Dubey et al., 1995a,b, 1996, 2015; Gamble et al., 2005; Hill et al., 2006; Gardner et al., 2010). The MAT has been extensively employed for detection of *T. gondii* antibodies in many species, including humans (Dubey, 2010).

In this study, we tested the serum of 3,275 participants by MAT, and the overall seroprevalence was 5.13%. These results were lower than those of national surveys of *T. gondii* conducted between 2000 and 2017 (8.20%) (Dong et al., 2018). This finding may be related to economic development and quality of life improvements, as people pay increasing attention to health and hygiene. In China, fully cooked food is more popular, and most people only drink boiled water and eat cooked meat, which greatly reduces the risk of *T. gondii* infection. The seroprevalence of *T. gondii* in humans in China is relatively low compared with other countries (Pappas et al., 2009; Dubey, 2010), a difference that may be explained by different dietary habits and other cultural habits. However, the seroprevalence of 5.13% in this survey indicated that humans from China are still widely exposed to *T. gondii*.

A significant increase in seroprevalence with age was demonstrated in this study, which is consistent with studies conducted in Egypt (Elsheikha et al., 2009), Northeast Brazil (Coêlho et al., 2003), and Nigeria (Kamani et al., 2009). This finding indicated that most *T. gondii* infections were obtained through postnatal transmission, and that accumulated exposure during a person's lifetime leads to an increased probability of infection.

There are only a few reports of seroprevalence in young children (Dubey, 2010). The maternal IgG antibodies to *T. gondii* can be transferred from a mother to baby through colostrum or the placenta (Gross et al., 2000; Miller et al., 2003). Usually, the passively transferred maternal IgG disappears by 12 months of age (Omata et al., 1994; Nielsen et al., 2005; Dubey, 2010). In this study, the prevalence of *T. gondii* IgG was 3.35% in nursing children, and two children (12 months) had high titers of 1:640 and 1:1280. Unfortunately, no follow-up studies have been conducted on nursing children, and we lack information about their mothers. It could not be ascertained if the *T. gondii* antibodies were passively transferred from the mothers or synthesized by the newborn child. However, congenital toxoplasmosis could not be ruled out in this survey.

In this study, human samples from five provinces were evaluated for *T. gondii* infection. We have summarized the available reports on human *T. gondii* infection from these provinces in Table 3. Here, the apparent seroprevalence was used to estimate the epidemiological regularity of *T. gondii* infection in humans. To obtain a clear picture of the true prevalence of *T. gondii* infection in China, in the future, the apparent seroprevalence needs to be estimated by Bayesian statistics for all unknown parameters (different sera, different serological test methods, and different test kits) (Basáñez et al., 2004). The present data were compared and interpreted to estimate the different levels of prevalence among similar populations. The total apparent seroprevalence of *T. gondii* was lower than of previous surveys (data from 2001 to 2017) (*p* > 0.05). In the Hubei province, the seroprevalence of *T. gondii* was significantly decreased compared to the survey results from 2001 to 2017 (*p* < 0.05). The difference may be related to the developed economy and hygiene, or to the limited number of samples (*n* = 300) collected from Hubei in this survey.

**Table 3:** Seroprevalence of *T. gondii* infection in humans from five provinces in China (2001–2017).

**Province**	**Sample Source**	**Method**	**Seropositivity (%)**	**(%) (/No. positive / No. tested)**	**This study**	**References**
Guangdong	Healthy person	IHA	10.10% (102/1010)	8.15% (379/4653)	8.26%↑ (80/968)	Guo et al., 2002
	Women, Slaughterer	ELISA	5.79% (22/380)			Lu et al., 2002
	Animal product processor, Animal breeders	ELISA	9.05% (21/232)			Zhao and Liu, 2007
	Resident	ELISA	5.56% (28/504)			Xie et al., 2004
	Resident	ELISA	8.16% (206/2526)			Feng et al., 2005
	Young students	MAT	0% (0/1)			Yang et al., 2017
Guangxi	Young students	MAT	3.85% (1/26)	3.85% (1/26)	4.80%↑ (13/271)	Yang et al., 2017
Shanghai	Veterinarian, Animal breeders, Resident	ELISA	6.57% (19/289)	4.09% (198/4839)	3.90%↓ (52/1334)	Wang et al., 2004
	Resident, Animal product processor, Animal breeders, Tumor patients	ELISA	4.03% (168/4169)			Ma et al., 2006
	Veterinarian, Animal product processor	ELISA	2.74% (10/365)			Chen et al., 2011
	Young students	MAT	6.25% (1/16)			Yang et al., 2017
Hubei	Pets breeder	ELISA	15.36% (155/1009)	7.39% (613/8294)	1.00%↓^*^ (3/300)	Chen, 2001
	Veterinarian, Animal breeders, Butcher Pregnant Women, Blood donor	ELISA	6.51% (141/3009)			Yu et al., 2007
	Women	ELISA	5.01% (51/1018)			Yin et al., 2005
	Resident	ELISA	8.21 (266/3240)			Zhu et al., 2013
	Young students	MAT	0% (0/18)			Yang et al., 2017
Shaanxi	Blood donor	ELISA	8.15% (30/368)	8.18% (32/391)	4.98%↓ (20/402)	Ai et al., 2007
	Young students	MAT	8.70% (2/23)			Yang et al., 2017
Total				6.72% (1223/18203)	5.13%↓ (168/3275)	

* *Statistically significant, the seroprevalence of T. gondii infection in humans in this study compared to previously studies*.

The seroprevalence of *T. gondii* in coastal areas (5.64%) was significantly higher than that in inland areas (3.28%). This may be explained by the subtropical monsoon climate, hot and humid climate, and lower altitudes in the coastal areas. Additionally, previous studies have shown that *T. gondii* oocysts can enter the ocean via rivers (Miller et al., 2002; Fayer et al., 2004; Dubey, 2010; Dong et al., 2018), that oocysts can be concentrated by filter-feeding invertebrates (Lindsay et al., 2001, 2004; Arkush et al., 2003), and that humans can be infected with *T. gondii* when they ingest undercooked shellfish or invertebrate predators. Furthermore, foreign food culture (undercooked steaks and vegetable salads) is more likely to affect residents of coastal areas, which tend to be more cosmopolitan than inland places.

According to the different geographical locations, we found that the seroprevalence of *T. gondii* in humans from south China was slightly higher than in the north, which is consistent with the results of Yang et al. (2017). The reason for the north–south difference is unclear, but it has been reported that *T. gondii* infection is more prevalent in warm climates and high rainfall areas than that in cold and dry areas (Dubey, 2010).

When a pregnant woman is primarily infected with *T. gondii*, the fetus may be infected through the placenta and even cause death (Nowakowska et al., 2005). In this study, the seroprevalence of *T. gondii* in pregnant women was 3.46%, within the range summarized by Gao et al. (2012), and lower than in some other countries such as Poland (40.6%), Ethiopia (68.4%) and Thailand (28.3%) (Nissapatorn et al., 2011; Nowakowska et al., 2012; Agmas et al., 2015). Compared with pregnant women, the seroprevalence of *T. gondii* in infertile women was 8.05%, and the difference was statistically significant. This finding is in agreement with others (Zhou et al., 2002; Dubey et al., 2014). There is indirect evidence indicating that chronic *T. gondii* infection may cause reproductive losses in small ruminants and mice (Dvorakova-Hortova et al., 2014; Hide et al., 2014). However, the relationship between chronic *T. gondii* infection and pregnancy problems in humans could not be firmly established, and more needs to be explored regarding human toxoplasmosis.

In addition, we found that men are more exposed to *T. gondii* infection than women; however, the differences were not significant. This result was consistent with the study by Yang et al. (2017), but contrary to the study by Xiao et al. (2010). Unfortunately, due to the lack of information, only a few participants were involved in the gender factor, so further investigation is necessary. The seroprevalence of *T. gondii* was higher in unhealthy people than in healthy people. This phenomenon was also found in humans and some animals (Tenter et al., 2000; Montoya and Liesenfeld, 2004; Dubey, 2008; Dong et al., 2018), which suggests that *T. gondii* infection may facilitate infection by other pathogens, or that unhealthy people may be more susceptible to *T. gondii*.

In addition, since the human sera were primarily collected from hospitals in this study, samples from asymptomatic humans was limited. More studies with larger sample sizes are necessary to confirm and extend the findings of this study.

## Conclusions

*Toxoplasma gondii* infection is reported in Chinese citizens in this study, and that higher age, living in coastal areas, and infertility were related to *T. gondii* infection. It is necessary to monitor the prevalence of *T. gondii* in food animals and felids, and powerful and effective regulatory measures should be undertaken to reduce human exposure to *T. gondii*. These include inactivating *T. gondii* oocysts, drinking boiled water, and eating well-cooked meat. More preventive measures should be initiated to reduce the infection rate of *T. gondii* in humans, such as the distribution of leaflets, by physicians and in health education classes containing recommendations on the nature of this disease and its avoidance.

## Data Availability Statement

The datasets generated for this study are available on request to the corresponding author.

## Ethics Statement

The studies involving human participants were reviewed and approved by Guideline for Using Subjects from Human and Animals by the Beijing Association for Science and Technology (SYXK [Beijing] 2007–0023). Written informed consent to participate in this study was provided by the participants' legal guardian/next of kin. Written informed consent was obtained from the individual(s), and minor(s)' legal guardian/next of kin, for the publication of any potentially identifiable images or data included in this article.

## Author Contributions

SX performed sample collection, laboratory tests, and contributed to the writing of the manuscript. RS and NJ performed sample collection and laboratory tests. LZ critically read and revised the manuscript. YY designed the study protocol, analyzed the results, and wrote the manuscript. All of the authors have read and approved the final version of the manuscript.

## Conflict of Interest

The authors declare that the research was conducted in the absence of any commercial or financial relationships that could be construed as a potential conflict of interest.
